# Two different protein expression profiles of oral squamous cell carcinoma analyzed by immunoprecipitation high-performance liquid chromatography

**DOI:** 10.1186/s12957-017-1213-5

**Published:** 2017-08-08

**Authors:** Soung Min Kim, Dasul Jeong, Min Keun Kim, Sang Shin Lee, Suk Keun Lee

**Affiliations:** 1 Department of Oral and Maxillofacial Surgery, School of Dentistry, Dental Research Institute, Seoul National University, Seoul, South Korea; 2 Department of Oral Pathology, College of Dentistry, Institute of Oral Science, Gangneung-Wonju National University, Gangneung, South Korea; 3 Department of Oral and Maxillofacial Surgery, College of Dentistry, Institute of Oral Science, Gangneung-Wonju National University, Gangneung, South Korea

**Keywords:** Protein expression profile, Oral squamous cell carcinoma (OSCC), Immunoprecipitation high-performance liquid chromatography (IP-HPLC), Potential target gene

## Abstract

**Background:**

Oral squamous cell carcinoma (OSCC) is one of the most dangerous cancers in the body, producing serious complications with individual behaviors. Many different pathogenetic factors are involved in the carcinogenesis of OSCC. Cancer cells derived from oral keratinocytes can produce different carcinogenic signaling pathways through differences in protein expression, but their protein expression profiles cannot be easily explored with ordinary detection methods.

**Methods:**

The present study compared the protein expression profiles between two different types of OSCCs, which were analyzed through immunoprecipitation high-performance liquid chromatography (IP-HPLC).

**Results:**

Two types of squamous cell carcinoma (SCC) occurred in a mandibular (SCC-1) and maxillary gingiva (SCC-2), but their clinical features and progression were quite different from each other. SCC-1 showed a large gingival ulceration with severe halitosis and extensive bony destruction, while SCC-2 showed a relatively small papillary gingival swelling but rapidly grew to form a large submucosal mass, followed by early cervical lymph node metastasis. In the histological observation, SCC-1 was relatively well differentiated with a severe inflammatory reaction, while SCC-2 showed severely infiltrative growth of each cancer islets accompanied with a mild inflammatory reaction. IP-HPLC analysis revealed contrary protein expression profiles analyzed by 72 different oncogenic proteins. SCC-1 showed more cellular apoptosis and invasive growth than SCC-2 through increased expression of caspases, MMPs, p53 signaling, FAS signaling, TGF-β1 signaling, and angiogenesis factors, while SCC-2 showed more cellular growth and survival than SCC-1 through the increased expression of proliferating factors, RAS signaling, eIF5A signaling, WNT signaling, and survivin.

**Conclusions:**

The increased trends of cellular apoptosis and invasiveness in the protein expression profiles of SCC-1 were implicative of its extensive gingival ulceration and bony destruction, while the increased trends of cellular proliferation and survival in the protein profile of SCC-2 were implicative of its rapid growing tumor mass and early lymph node metastasis. These analyses of the essential oncogenic protein expression profiles in OSCC provide important information for genetic counseling or customized gene therapy in cancer treatment. Therefore, protein expression profile analysis through IP-HPLC is helpful not only for the molecular genetic diagnosis of cancer but also in identifying target molecules for customized gene therapy in near future.

## Background

Squamous cell carcinoma (SCC) is the most frequent and serious malignant tumor in the oral cavity. Although it can be easily recognized by patients themselves or detectable through simple clinical observation, the surgical removal of a SCC lesion is still difficult due to complicated anatomical structures in the oral and maxillofacial region composed of neuromuscular and dento-skeletal tissues [1, 2]. Therefore, the combination treatment of surgery, radiation, and chemotherapy is frequently recommended following a pathological diagnosis. Surgical therapy is the immediate and primary treatment to eradicate the cancer tissue, and radiation therapy and chemotherapy have been developed to induce cellular apoptosis and arrest the growth of cancer cells [3–5].

Many cases of oral squamous cell carcinoma (OSCC) recur even after radical excision of the tumor lesion, leading to a poor prognosis. Radiation therapy and chemotherapy are also sometimes ineffective on target cancer cells depending on their oncogenic status in terms of cellular differentiation, proliferation, apoptosis, survival, migration, and other factors. In order to properly treat OSCCs, pathological examination should be carefully performed along with a molecular biological investigation to determine the final diagnosis. Therefore, it is critical to determine the cellular biological status of cancer cells, which could be identified by their protein expression profiles for different oncogenic signaling pathways [6, 7]. The present study was performed to explore the molecular biological dynamics in two different types of OSCCs through analysis with immunoprecipitation high-performance liquid chromatography (IP-HPLC).

The two OSCCs showed different histological features in cancer growth and propagation and had contrary protein expression profiles implicative of their differences in carcinogenesis progression. Fortunately, a sufficient amount of protein extract was obtained from both cases of OSCCs during surgical excision of the tumor mass followed by selective neck dissection. Each protein extract was analyzed through IP-HPLC methods, which have been much improved in data accuracy and statistical analysis. These results are discussed along with a review of the literature.

## Methods

Two representative OSCCs were selected from the files of the Department of Oral Pathology, Gangneung-Wonju National University Dental Hospital (GWNUDH) under the approval of institutional review board (IRB2016–11). Both cases occurred in a mandibular molar (SCC-1) and posterior maxillary area (SCC-2) in male patients who were 65 and 69 years old. However, their clinical/radiological features and pathological diagnosis were somewhat different, as was their subsequent prognosis.

### Patients data

**Fig. 1:**
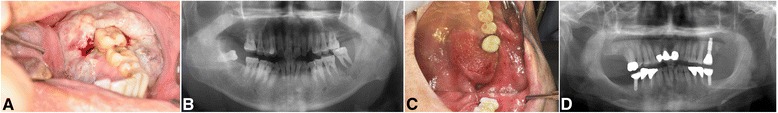
Clinical and panoramic views of SCC-1 (**a**, **b**) and SCC-2 (**c**, **d**). A large gingival ulceration (**a**) with extensive bony destruction (**b**) in the left posterior mandible of SCC-1, and a relatively small papillary gingival swelling (**c**) with bony destruction (**d**) in the left posterior maxilla of SCC-2

SSC-1 case showed more extensive bony destruction around the mandibular molar area involving the upper half of the mandibular body. The patient is a 65-year-old man with large gingival ulceration with severe halitosis on the lower left gingiva, 4 × 6 cm sized with extensive buccal cortex bony invasion. It was diagnosed as a squamous cell carcinoma with stage IV, thus partial mandibulectomy with functional neck dissection with level I to III, reconstruction with an R-plate and radial forearm free flap. Post-operative radiotherapy with 7200 Gray, and no recurrence or metastasis during 3 years and 6 months’ follow-up period (Fig. 1, Table 1).

**Table 1:** Clinical courses of SCC-1 and SCC-2

Patient	Age	Sex	Size (cm)	Location	Stage	Adjuvant therapy	Operation	Follow-up	Recurrence
SCC-1	65	M	4 × 6	Lt Mn	pT4N0M0	PORT	Partial mandibulectomy, SOHND, R-plate with RFFF reconstruction	3 years and 6 months	None
SCC-2	69	M	2 × 3	Lt Mx	pT3aN0M0	PORT	Extended maxillectomy, SOHND, local flap with buccal fat graft	4 years	Neck metastasis

*SCC* squamous cell carcinoma, *Lt* left, *Mx* maxilla, *Mn* mandible, *PORT* post-operative radiation therapy, *SOHND* supraomohyoid neck dissection, *RFFF* radial forearm free flap

SCC-2 case showed localized bony destruction around the left maxillary posterior gingiva and early tumor metastasis to the cervical lymph nodes. The patient was a 69-year-old man with a relatively small papillary gingival swelling mass on the left upper posterior gingiva. This lesion grew rapidly to form a 4 × 6 cm sized submucosal mass and was diagnosed as a squamous cell carcinoma with stage III. Extended maxillectomy with functional neck dissection of level I to III combined with buccal fat graft were operated. Although post-operative radiotherapy with 6500 Gray was executed, there was cervical node metastases during 4 years’ follow-up period (Fig. 1, Table 1). Unfortunately, the patient is not followed-up anymore.

### Histological and immunohistochemical staining

The surgically removed specimens were fixed in 10% neutral buffered formalin, processed routinely, and embedded in paraffin. Histologic sections with a thickness of 4 μm were mounted on glass slides and stained with hematoxylin and eosin. Serial micro-sections were also prepared for immunohistochemical staining using the different antisera listed in Table 2. The immunohistochemical reaction protocols used for this study differed according to the target antigen and manufacturers’ protocols. Briefly, after deparaffinization and rehydration of the tissue sections in xylene followed by ethanol, sections were incubated with 0.5% hydrogen peroxide in phosphate-buffered saline for 30 min. Primary anti-human (rabbit/mouse/goat) polyclonal antibodies were applied to each micro-section using the triple sandwich indirect immunohistochemical method [8]. Microscopic images were captured by a digital camera (DP-70®, Olympus Co., Japan), followed by statistical analysis using the image analysis program (IMT i-Solution®, ver 21.1, Vancouver, Canada).

**Table 2:** Antibodies used in this study

Signaling proteins	Number	Antibodies
Cytoskeletal proteins	1	α-Tubulin^a^
Growth factor-related proteins	5	EGFR^b^, c-erbB2^b^, TGF-β1^d^, bFGF^a^, HGF^b^
Proliferation-related proteins	9	eIF5A^c^, DHS^c^, DOHH^c^, PCNA^c^, MPM-2^b^, CDK4^a^, cMyc^a^, MAX^a^, hTERT^a^
Transcription signaling proteins	3	NFkB^b^, p38^a^, E2F-1^a^
Apoptosis-related proteins	14	FAS^a^, FASL^a^, PARP^a^, BAX^a^, NOXA^a^, PUMA^a^, BAD^a^, BAK^a^, BID^a^, caspase 3^a^, caspase 8^a^, caspase 9^a^, FADD^a^, FLIP^a^,
Cell survival-related proteins	3	pAKT^c^, MDM2^a^, BCL2^a^
Tumor suppressor proteins	8	p16^a^, p21^a^, p53^a^, p63^a^, RB1^a^, PTEN^a^, PTCH^a^, NF-1^b^
Oncoproteins	9	14-3-3^a^, CEA^c^, STAT3^a^, survivin^d^, DMBT1^a^, maspin^a^, snail^a^, KRAS^c^, PIM1^a^
Protection proteins	5	HO-1^a^, caveolin^a^, HSP-70^a^, FAK^a^, TGase-1^b^
Proinflammation proteins	2	TNFα, SHP-1
WNT/β-catenin pathway proteins	4	SHH^a^, β-catenin^b^, WNT1^a^, APC^a^
Matrix proteolysis proteins	4	MMP-1^c^, MMP-2^c^, MMP-9^a^, elaffin^a^
Angiogenesis-related proteins	5	HIF^d^, VEGF^d^, vWF^c^, angiogenin^a^ **,** CMG2^b^
Total	72	

Abbreviation: *pAKT* v-akt murine thymoma viral oncogene homolog (phosphorylated at Thr 308), *APC* adenomatous polyposis coli, *BAD* BCL2 associated death promoter, *BAK* BCL2 antagonist/killer, *BAX* BCL2 associated X, *BCL-2* B-cell leukemia/lymphoma-2, *BID* BH3 interacting-domain death agonist, *CDK4* cyclin dependent kinase 4, *CEA* carcinoembryonic antigen, *CMG2* capillary morphogenesis protein 2, *DHS* deoxyhypusine synthase, *DOHH* deoxyhypusine hydroxylase, *DMBT1* deleted in malignant brain tumors 1, *E2F-1* transcription factor, *EGFR* epithelial growth factor receptor, *eIF5A* eukaryotic translation initiation factor 5A, *FADD* FAS associated via death domain, *FAK* focal adhesion kinase, *FAS* CD95/Apo1, *FASL* FAS ligand, *bFGF* basic fibroblast growth factor, *FLIP* FLICE-like inhibitory protein, *HGF* hepatocyte growth factor, *HIF* hypoxia inducible factor, *HO-1* hemoxygenase 1, *HSP-70* heat shock protein-70, *KRAS* V-Ki-ras2 Kirsten rat sarcoma viral oncogene homolog, *MAX* myc-associated factor X, *MDM2* mouse double minute 2 homolog, *MMP-1* matrix metalloprotease-1, *MPM-2* mitotic protein monoclonal 2, *cMyc* V-myc myelocytomatosis viral oncogene homolog (avian), *NF-1* neurofibromin-1, *NFkB* nuclear factor kappa-light-chain-enhancer of activated B cells; *NOXA* phorbol-12-myristate-13-acetate-induced protein 1; *PARP* poly-ADP ribose polymerase, *PCNA* proliferating cell nuclear antigen, *PIM1* pivotal integration site 1, *PTCH* patched homolog, *PTEN* phosphatase and tensin homolog, *PUMA* p53 up-regulated modulator of apoptosis, *RB1* retinoblastoma 1, *SHH* sonic hedgehog, *SHP-1* short helical protein-1, *SOS-1* Son of sevenless-1, *STAT3* signal transducer and activator of transcription-3, *hTERT* human telomerase reverse transcriptase, *TGase-1* transglutaminase-1, *TGF-β1* transforming growth factor-β1, *TNFα* tumor necrosis factor-α, *VEGF* vascular endothelial growth factor, *vWF* von Willebrand factor

^a^Santa Cruz Biotechnology, USA

^b^DAKO, Denmark

^c^Neomarkers, CA, USA

^d^ZYMED, CA, USA

### IP-HPLC analysis for the protein extract obtained from RAW 264.7 cell culture

One hundred microgram of each protein extract was applied to the immunoprecipitation procedure using a protein A/G agarose column (Amicogen Co., Korea). The protein A/G agarose columns were separately pre-incubated with 1 μg of each of the 25 different antisera, including β-actin, Ki-67, PCNA, MAX, cMyc, E2F-1, Rb-1, and MAD (Santa Cruz Biotech, USA). Briefly, the protein samples were mixed with 5 mL of binding buffer (150 mM NaCl, 10 mM Tris pH 7.4, 1 mM EDTA, 1 mM EGTA, 0.2 mM sodium vanadate, 0.2 mM PMSF, and 0.5% NP-40) and incubated in the protein A/G agarose columns at 10 °C for 1 h. The columns were placed on a rotating stirrer during the incubation. After washing each column with a sufficient amount of PBS solution (pH 7.3, 137 mM NaCl, 2.7 mM KCl, 43 mM Na_2_HPO_4_-7H_2_O, and 1.4 mM KH_2_PO_4_), the target protein was eluted with 150 μL of IgG elution buffer (Pierce Co., USA). The immunoprecipitated proteins were analyzed by HPLC (1100 series®, Agilent, USA) using a reverse phase column and micro-analytical detector system (SG Hightech Co., Korea), operated with a 0.15 M NaCl, 20% acetonitrile solution at 0.4 mL/min for 30 min, and analyzed by UV spectroscopy at 280 nm. IP-HPLC analysis was performed simultaneously for both the control and experimental groups.

In the IP-HPLC results, the sample protein peak areas (mAU*s) obtained from HPLC analysis in the negative control were used to eliminate the antibody peak area (mAU*s) [9, 10]. To compare the two different types of SCCs, the protein peak area values of SSC-1 and SSC-2 were proportionally normalized by the α-tubulin value and plotted as a bar and radial line graph.

## Results

### Histological and immunohistochemical findings

**Fig. 2:**
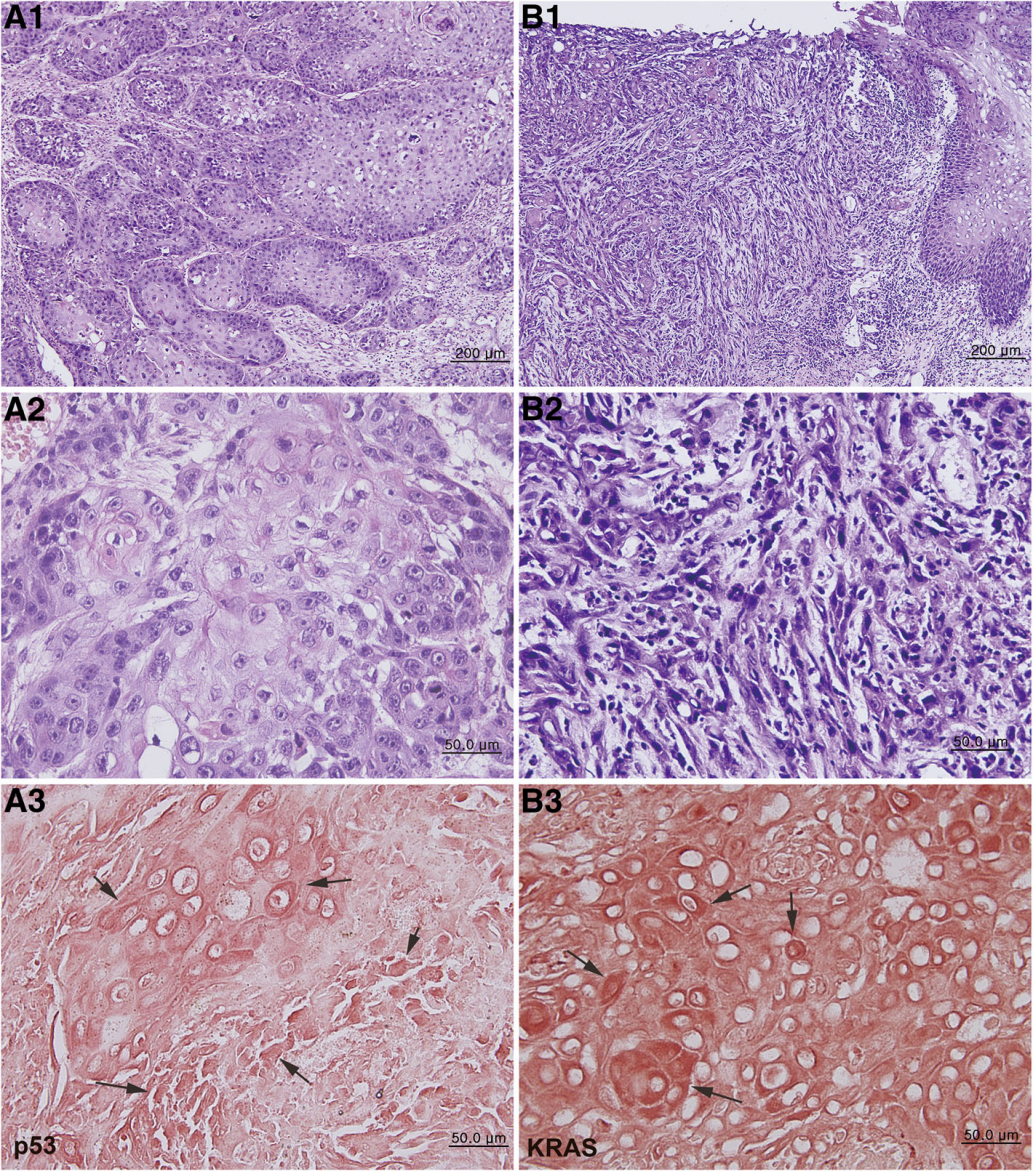
Photomicrographs of two different types of OSCCs. *A1*-*A3*: SSC-1, well differentiated with many cancer pearls. *B1*-*B3*: SSC-2, poorly differentiated with numerous infiltrating tumor islets. *A1*, *A2*, *B1*, *B2*: hematoxylin and eosin staining. *A3* and *B3*: Immunostaining without background stain. *A3*: p53 staining is strongly positive in the tumor cells (*arrows*). *B3*: KRAS staining is strongly positive in the tumor cells (*arrows*)

Histologically, SSC-1 was diagnosed as a well differentiated SCC forming many cancer pearls (Fig. 2 A1-A2), and SCC-2 was diagnosed as a poorly differentiated OSCC exhibiting numerous infiltrating tumor islets into the underlying connective tissue (Fig. 2 B1-B2).

In the immunohistochemical staining, the SSC-1 tumor cells were strongly positive for p53 (Fig. 2 A3), TGF-β1, c-erbB2, caspase-9, PARP, FAS, FASL, MMP-2, and MMP-9, while the SSC-2 tumor cells were strongly positive for KRAS (Fig. 2 B3), STAT3, MPM2, eIF5A, DHS, DOHH, snail, and survivin (data not shown).

### IP-HPLC analysis from SCC-1 and SCC-2

**Fig. 3:**
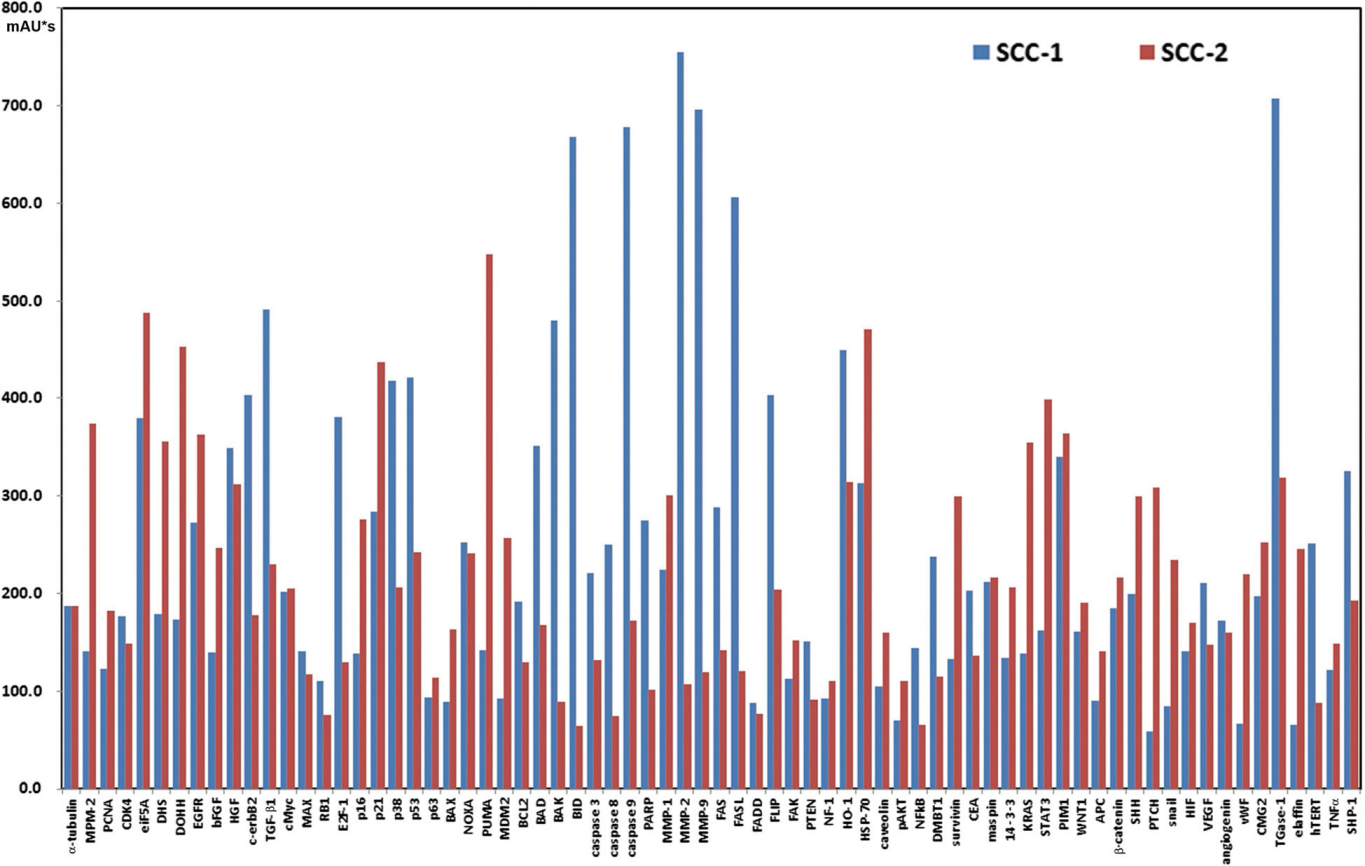
A bar graph comparing the essential oncogenic protein expression profiles between the two different types of oral squamous cell carcinomas. SCC-1 (*blue*) showed more cellular transformation and apoptosis than SCC-2 (*red*) by the overexpression of caspases, MMPs, p53 signaling, FAS signaling, TGF-β1 signaling, and angiogenesis factors, while SCC-2 showed more invasive growth and cellular survival than SCC-1 by the overexpression of proliferating factors, RAS signaling, eIF5A signaling, Wnt signaling, and survivin

**Fig. 4:**
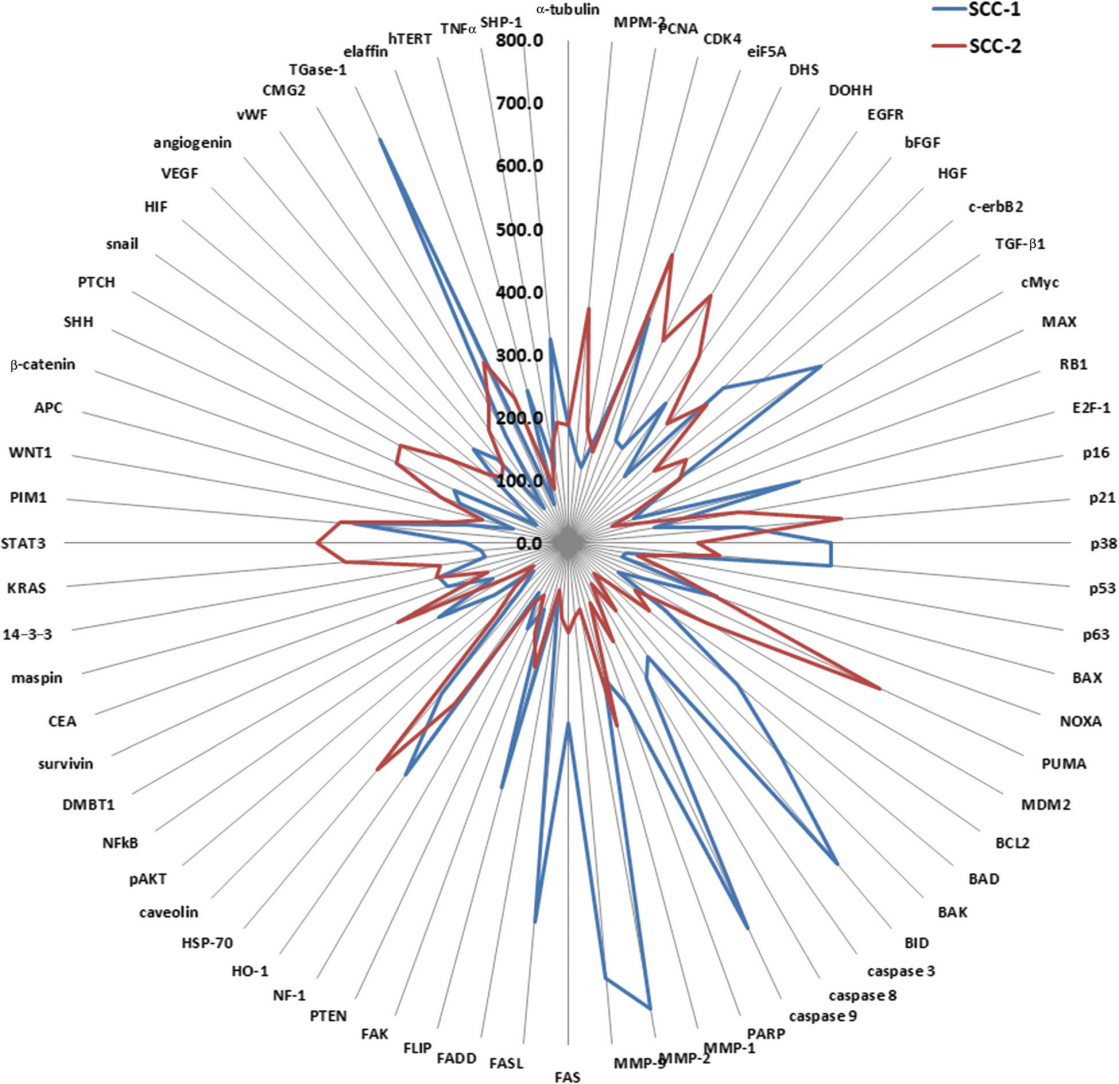
A radial line graph demonstrating the differential expression of essential oncogenic protein groups between SCC-1 (*blue*) and SCC-2 (*red*) using the same data as in Fig. 3

The IP-HPLC analysis revealed that SCC-1 showed more cellular transformation and apoptosis than SCC-2, while SSC-2 showed more invasive growth and cellular survival than SCC-1 (Figs. 3 and 4). In the protein expression profile of SSC-1, the neoplastic proliferation of tumor cells was supported by the overexpression of E2F-1 and c-erbB2, and the cellular transformation and differentiation of tumor cells were related to the overexpression of TGF-β1, TGase-1, HO-1, hTERT, and p38 compared to SSC-2. Particularly, SSC-1 showed overexpression of apoptosis-related proteins, e.g., p53, BAD, BAK, BID, BCL2, FAS, FASL, FLIP, caspase-3, caspase-8, caspase-9, and PARP compared to SSC-2, indicating that the oncogenic progression in SSC-1 was related to the activation of p53 and FAS signaling compared to SSC-2 (Fig. 3).

On the other hand, in the protein expression profile of SSC-2, the neoplastic proliferation of the tumor cells was related to the overexpression of PCNA, MPM2, KRAS, STAT3, EGFR, and bFGF and supported by the overexpression of protein translation factors, e.g., eIF5A, DHS, and DOHH, compared to SSC-1. The p53 expression was partly suppressed by the overexpression of MDM2, followed by the compensatory overexpression of p16 and p21. The oncogenic progression was relevant to the activation of RAS and WNT signaling proteins, e.g., KRAS, STAT3, WNT1, β-catenin, snail, and PTCH, compared to SSC-1. The tumor cells also showed increased cellular survival by the overexpression of survivin, HSP-70, 14–3-3, and angiogenesis-related proteins, e.g., HIF, vWF, CMG2, and bFGF, compared to SSC-1 (Fig. 3).

The radial line graph shown in Fig. 4 clearly demonstrates the differences in essential protein expression profiles between SSC-1 and SSC-2. The protein expression of SSC-1 was shifted into the abortive cycles of cellular differentiation, transformation, and apoptosis, while the protein expression of SSC-2 was shifted into the abortive cycles of oncogenic cellular growth and survival (Fig. 4). These findings indicate that the carcinogenesis progression processes of the two SCCs are different even though they are both derived from keratinocytes of the oral mucosa.

## Discussion

The present study investigated the protein expression profiles of two representative types of OSCCs. Although these data were obtained from preliminary analysis in a series of OSCC research, the recent strategy of molecular biological gene therapy urgently recommends the collection of oncogenic signaling data from cancer cells in each individual patient. Therefore, our study utilized IP-HPLC analysis, which has been designed to perform quantitative protein analysis using different but comparable protein samples.

During the past several years, liquid chromatography tandem mass spectrometry (LC-MS/MS) has emerged as an innovative analytical technology applicable to wide ranges of sample’s molecules. Mass spectrometry (MS) is an analytical technique that ionizes chemical species and sorts the ions based on their mass-to-charge ratio, and the mass spectrum is a plot of the ion signal as a function of the mass-to-charge ratio. These spectra are used to determine the elemental or isotopic signature of a sample, the masses of particles and of molecules, and to elucidate the chemical structures of molecules, such as peptides and other chemical compounds [11]. MS has increased in speed, accuracy and use, and with the ability of the mass spectrometers to identify increasing numbers of proteins, the identification of undesirable peptides has also increased [12]. Because the IP-HPLC analysis is based on the antibody interaction with target protein which may be specific and sensitive depending on the epitope binding activity of antibody, it has to utilize mathematical calculation for the relative protein quantitation compared to the control. Therefore, it is thought that the data obtained from IP-HPLC analysis may be quite different from those from mass spectrometry (MS-MS) which is able to provide the absolute quantitation of proteins.

The histological differences between SSC-1 and SSC-2 were characterized by the dominant carcinogenic features of cellular proliferation, apoptosis, invasion, and survival, which were related to differences in oncogenic signaling in cancer cells. In the immunohistochemical staining, the tumor cells of SSC-1 were strongly positive for p53, TGF-β1, c-erbB2, caspase-9, PARP, FAS, FASL, MMP-2, and MMP-9, while the tumor cells of SSC-2 were strongly positive for KRAS, STAT3, MPM2, eIF5A, DHS, DOHH, snail, and survivin. These findings were similar to many previous reports [13–18] illustrating how oncogenic signaling functions in cancer cells, although their expression levels were not quantitative but derived from the intensity of peroxidase reaction with the chromogens 3,3′-diaminobenzidine (DAB) or 3-amino-9-ethylcarbzole (AEC). Therefore, a more precise detection system should be applied in the investigation of protein expression for molecular signaling as in this study.

The present IP-HPLC analysis disclosed that the neoplastic proliferation of SCC-1 was related to the overexpression of E2F-1 and c-erbB2, and the cellular transformation and differentiation of SCC-1 was related to the overexpression of TGF-β1, TGase-1, HO-1, hTERT, and p38 compared to SSC-2. Particularly, SSC-1 showed the overexpression of apoptosis-related proteins, e.g., p53, BAD, BAK, BID, BCL2, FAS, FASL, FLIP, caspase-3, caspase-8, caspase-9, and PARP, compared to SCC-2, indicating that oncogenic progression in SSC-1 is related to the activation of p53 and FAS signaling or cellular apoptosis compared to SSC-2. Thus, oncogenic signaling could progress from multiple pathways involved in cellular proliferation, differentiation, apoptosis, and survival in cancer cells, and these results were similar to those of previous reports [19–21].

On the other hand, in the protein expression profile of SSC-2, the neoplastic proliferation of tumor cells was related to the overexpression of PCNA, MPM2, KRAS, STAT3, EGFR, and bFGF and was supported by the overexpression of the protein translation factors eIF5A, DHS, and DOHH compared to SSC-1. Therefore, it was presumed that the major oncogenic signaling of SSC-2 was derived from RAS signaling supported by different growth factors and active protein translation [22–24].

The p53 expression in SSC-2 was partly suppressed by the overexpression of MDM2, followed by the compensatory overexpression of p16 and p21; thereby, the major tumor suppressor protein p53 might be down-regulated and compensated by other cell cycle inhibitors in SCC-2. Through comparison of the protein expression profiles of SCC-1 and SCC-2, the oncogenic progression of SSC-2 was assumed to be related to activation of the RAS and WNT signaling proteins, e.g., KRAS, STAT3, WNT1, β-catenin, snail, and PTCH, compared to SSC-1 [25]. Therefore, the propagation of SSC-2 was more aggressive with early cervical lymph node metastasis and rapid recurrence compared to SSC-1 even after radical surgery.

The tumor cells of SSC-2 also showed increased cellular survival by the overexpression of survivin [26], HSP-70, 14-3-3, and the angiogenesis-related proteins HIF, vWF, CMG2, and bFGF compared to SSC-1 [27]. It was presumed that cellular protection, survival, and angiogenesis are closely associated with each other and support or compensate their molecular signaling, resulting in propagation of cancer cells. Therefore, these signaling pathways could be oncogenic for SCC as well as potentially important proteins for targeting by anti-cancer drugs.

The radial line graph (Fig. 4) clearly demonstrates the differences in essential protein expression profiles between SSC-1 and SSC-2. The protein expression of SSC-1 was shifted into the abortive cycles of cellular differentiation, transformation, and apoptosis, while the protein expression of SSC-2 was shifted into the abortive cycles of oncogenic cellular growth and survival. These findings indicate that the carcinogenesis progression of these two SCCs are contrary even though they are both derived from keratinocytes of the oral mucosa.

As cellular apoptosis was dominant in the oncogenic signaling of SCC-1 with the overexpression of p53, it is suggested that SCC-1 could be effectively treated by radiation therapy, which can induce severe DNA damage followed by cellular apoptosis. For SCC-2, which showed dominant expression of RAS and WNT signaling, it is suggested to treat with multiple drugs targeting the RAS and WNT pathways and related proteins. For the “apoptosis-related proteins” in Table 2, the regulation of apoptosis and cell proliferation by oncogenes, tumor-suppressor genes and growth factors in OSCC was already well known in many previous published articles [28–30]. About the p53, TNF, and Fas signaling in apoptosis, two theories of the direct initiation of apoptotic mechanisms in mammals have been suggested (https://en.wikipedia.org/wiki/Fas_ligand and https://en.wikipedia.org/wiki/Apoptosis). The TNF-induced model and the Fas-Fas ligand-mediated model, both involving receptors of the TNF receptor (TNFR) family coupled to extrinsic signals. Fas ligand (FasL or CD95L) is a type II transmembrane protein that belongs to the TNF family. Its binding with its receptor induces apoptosis. Fas ligand/receptor interactions play an important role in the regulation of the immune system and the progression of cancer, including OSCC.

The present study is a simple demonstration of the comparison of oncogenic protein expression profiles between different types of OSCCs, indicating that further investigation should be performed by examining more cases of OSCCs using precise molecular biological methods. However, it is highly recommended that various anti-cancer drugs be developed in order to target specific oncogenic proteins in contrast to conventional chemotherapy using aggressive alkylating agents such as cisplatin, 5-fluorouracil, and methotrexate.

## Conclusions

The increased trends of cellular apoptosis and invasiveness in the protein expression profile of SCC-1 implicated its extensive oral ulceration and bony destruction, while the increased trends of cellular proliferation and survival in the protein profile of SCC-2 supported its rapid growing tumor mass and early lymph node metastasis. These analyses of essential oncogenic protein expression profiles in OSCCs provide important information for genetic counseling or customized gene therapy in cancer treatment.
